# Society Meetings

**Published:** 1899-03

**Authors:** 


### SOCIETY MEETINGS.

THE American Academy of Medicine will hold its next annual meeting at Columbus, O., June 3-5, 1899. The preliminary program includes thus far, the following papers arranged under the special topic to be discussed.

*Specialism in Medicine.*

1. The ethics of specialism, by Dr. A. L. Benedict, Buffalo.

2. How far has specialism benefited the ordinary practice of medicine, by Dr. L. Duncan Bulkley, New York.

3. The relation of specialism to general medicine, by Dr. Solomon Solis-Cohen, Philadelphia, Pa.

4. The specialties and commercialism, by Dr. H. Bert Ellis, Los Angeles, Cal.

5. Subject to be announced. By Dr. G. Hudson Makuen, of Philadelphia, Pa.

6. Specialists and therapy, by Dr. Elmer Lee, of New York.

7. Specialism in its relation to other branches of medicine, by Dr. G. W. McCaskey, of Fort Wayne, Ind.

*Advertising and the Medical Profession.*

1. Medical advertising, by Dr. Robert H. Babcock, of Chicago.

2. The ethics of advertising applied to the medical profession, by Dr. A. Ravogli, of Cincinnati.

3. Subject to be announced. By Dr. J. A. Lichty, of Clifton Springs, N. Y.

*The Medical Service of the Army and Navy.*

1. Aspects of the late war from a naval surgeon's standpoint, by Dr. W. C. Braisted, U. S. N.

2. Subject to be announced. By Dr. G. G. Groff, now in Puerto Rico.

3. What should be aimed at in a reorganisation of the national guard system, so far as its medical department is concerned? by Dr. C. B. Nancrede, late of the U. S. V., of Ann Arbor, Mich.

4. The relation of the medical profession to the army and navy, as developed in the Spanish-American war, by Dr. James A. Pilcher, U. S. A.

*Miscellaneous Papers.*

1. Remarks on hospital organisation, with special reference to continuous service, by Dr. J. C. Wilson, of Philadelphia.

*Papers on Subjects Not Yet Furnished.*

1. By Dr. Edward Jackson, of Denver, president's address.
2. By Dr. J. R. Smith, U. S. A. Retired.
3. By Dr. V. C. Vaughan, of Ann Arbor.
4. By Dr. C. T. McClintock, of Detroit.
5. By Dr. A. J. Puls, of Milwaukee.

The secretary, Dr. Charles McIntire, Easton, Pa., should be addressed for any information in relation to the meeting.

---

THE Buffalo Academy of Medicine held meetings during the months of January and February, 1899, as follows:

*Section on Obstetrics.*—Tuesday evening, January 31st, program: Report of 170 obstetric cases, Dr. C. A. Clements. Melena Monatorum, Dr. C. C. Frederick.

Tuesday evening, February 28th, program: Some remarks on occiputo posterior positions, Dr. Van Peyma. Discussion by Drs. Mann, Crockett, Hayd, Bank, Frederick, Howell.

*Surgical Section.*—Tuesday evening, February 7th, program: Stricture of the lachrymal duct; treatment by electrolysis and otherwise, Dr. B. H. Grove. Discussion by Drs. Renner, Henkel, Blaauw. The treatment of hernia without operation, Dr. Wm. C. Phelps. Discussion by Drs. E. A. Smith, Dooley, York, Thompson and others.

*Section on Medicine.*—Tuesday evening, February 14th, program: The status of pharmacy today and its possible improvements, Dr. Eli H. Long. Discussion by Drs. Allen A. Jones, Willis G. Gregory, G. W. York, H. C. Buswell, Mr. George Reimann, Mr. J. A. Lockie.

*Section on Pathology.*—Tuesday evening, February 21st, program : Intraocular tumors, Dr. B. H. Grove; Pathology of alcoholism, Dr. J. W. Grosvenor. Discussion opened by H. R. Hopkins.

---

The Tri-state Medical Association of Mississippi, Arkansas and Tennessee, held its regular annual meeting at Memphis, December 20, 21 and 22, 1899, at which the following resolutions were adopted :

Whereas, the medical laws of the various states have been so perverted by political influences as to give legislative sanction to grotesque, ignorant and dangerous sects of pretenders and charlatans ; and

Whereas, The privileges granted to one of the most outrageous aberrations—namely, the so-called osteopathy, constitute a disgrace to the states in which the "osteopathists" are legally entrenched; and

Whereas, A certain William Smith, osteopathist, having been roundly denounced, together with his sect, by Parke, Davis & Co., and the *Medical Age*, now brings suit against both for $25,000 damages; therefore,

Be it declared the sentiment of the Tri-State Medical Association, of Mississippi, Arkansas and Tennessee, that Parke, Davis & Co. and the *Medical Age* are entitled to the sympathy of its members and of all medical practitioners; that we wish and expect them to enjoy a complete triumph in repelling this legal assault, and that wheresoever a powerful house takes a bold stand in opposition to quackery it promotes the interests of legitimate and honorable medicine and the welfare of humanity.

---

The preliminary program of the seventh International congress against the abuse of alcoholic liquors, to be held in Paris, April 4 to 9, 1899, has been issued and it promises to be the widest discussion of this topic ever made at any one gathering. Nearly every aspect or phase of alcoholic injury and loss is treated by persons familiar with and able to discuss it. The morning sessions are to be confined to scientific studies. It is proposed to have representatives of every society and organisation for temperance in the world, and if possible arouse a new interest in this topic, which is fast becoming the great theme of civilisation. Twelve great countries of Europe have announced delegates to this meeting, and nearly a hundred leading men have been enrolled to take part. Five hundred delegates are expected, and it is anticipated that this meeting will be one of the most memorable ever held devoted to this subject. Short papers are earnestly solicited on any one phase of the subject from American workers in this field. All letters should be addressed to the American chairman of the organisation, T. D. Crothers, M. D., Hartford, Conn.

---

THE Lake Erie Medical Society will hold its next regular meeting at Dayton, N. Y., March 3, 1899. The following program is announced: Albuminuria in pregnancy, W. J. French, Hamlet, N. Y.; A plea for ye olden remedies, E. E. Davis, Forestville, N. Y. Presentation of cases. Intussusception, William Robert Henderson, Buffalo, N. Y.; Hemorrhoids, F. C. Beals, Salamanca, N. Y.; Enteric fever, J. Cherry, Eden, N. Y.

The officers are: President, W. W. Jones, Dayton, N. Y.; vice-president, B. S. Bourne, Hamburg, N. Y.; secretary and treasurer, S. S. Bedient, Little Valley, N. Y.; program committee, W. W. Jones, Dayton, N. Y.; S. S. Bedient, Little Valley, N. Y.

---

THE Cincinnati Obstetrical Society at its last annual meeting elected as officers for the ensuing year: President, Dr. Edwin Ricketts; vice-president, Dr. Julia Carpenter; secretary, Dr. William Gillespie; treasurer, Dr. M. A. Tate.

---

THE official circular has been sent out announcing the meeting of the ninth International Ophthalmological Congress, which will be held at Utrecht, Holland, August 14 to 18, 1899.

---

THE Esculapian Club, of Buffalo, will at its March meeting be entertained by Dr. C. B. LeVan, at his residence, 1298 Jefferson street. Dr. F. J. Carr is the essayist.

